# Are Conference Posters Being Cited?

**DOI:** 10.3389/frma.2021.766552

**Published:** 2021-11-26

**Authors:** Nick Haupka, Cäcilia Schröer, Christian Hauschke

**Affiliations:** ^1^ Faculty 4: Mathematics, Natural Sciences, Economics and Computer Science, University of Hildesheim, Hildesheim, Germany; ^2^ TIB – Leibniz Information Centre for Science and Technology, Hannover, Germany; ^3^ Faculty of Media, Information and Design, University of Applied Sciences and Arts Hannover, Hannover, Germany

**Keywords:** academic posters, citations, citation analysis, scholarly communication, conferences

## Abstract

We present a small case study on citations of conference posters using poster collections from both Figshare and Zenodo. The study takes into account the years 2016–2020 according to the dates of publication on the platforms. Citation data was taken from DataCite, Crossref and Dimensions. Primarily, we want to know to what extent scientific posters are being cited and thereby which impact posters potentially have on the scholarly landscape and especially on academic publications. Our data-driven analysis reveals that posters are rarely cited. Citations could only be found for 1% of the posters in our dataset. A limitation in this study however is that the impact of academic posters was not measured empirical but rather descriptive.

## Introduction

The output that is analyzed in scientometric analysis oftentimes consists of conventional publication types like academic articles, monographs or papers in conference proceedings. Recently, the research about the impact of research software and research data has seen some growth. It is remarkable that another traditional means of scholarly communication, the academic poster, barely gets any attention when it comes to scientometric analysis (Rowe 2017a). Hence, this study analyzes whether or not academic posters are cited. To do this, it is necessary to distinguish what a scientific poster represents in comparison to other mediums of scholarly communication.

Usually, academic posters are a visual instrument to illustrate research ideas in a poster session at an academic conference (McCann et al., 1994). In contrast to reading research articles, the purpose of poster sessions is to attract and connect with students, colleagues and professionals on a specific research topic or special field of interest through a poster (Sexton 1984; Moneyham et al., 1996). In addition, poster presentations can be seen as a method of assessment which may help students and researchers to improve their practices and techniques (Bracher 1998). Poster exhibitions are also an effective form of the exchange of concepts, views and opinions at conferences (Schmidmaier 1981). Although posters are primarily a visual medium, they do require communication skills to provide exceeding information on the poster to outsiders (Rowe and Ilic, 2008). The average poster consists of four sections, namely, introduction, method, results and discussion which shows similarities to the structures of journal abstracts and scientific experiments (Shalom 1993; Rowe 2017b). Like journal abstracts, each poster merely gives information on the purpose of the study carried out by the researcher, a description of the used tools and procedures, a major conclusion as well as implications for research (Sexton 1984). Normally, these information are enriched with pictures and other visually stimulating elements to attract the attention of conference attendees.

Investigations on the effects and benefits of scholarly posters have existed for many decades now. First attempts to summarize and assess the influence of posters were undertaken in the mid 1970s. Maugh (1974) wrote that poster sessions are in many respects “a better way” of communication in contrast to reading and presenting full text papers at conferences. For instance, poster sessions give speakers more time to explain their research findings in-depth in comparison to conventional presentations. Further studies supported the idea of presenting research findings in the form of a poster and came to the conclusion that poster sessions can be a substitution and alternative to the traditional approach of reading research articles (Sexton 1984). It is estimated that between 1969 and 2014 poster presentations at the Federation of European Biochemical Societies (FEBS) meetings have become up to 40 times more frequent (Rowe and Ilic, 2015). Also, since the 1990s an exponential increase of posters and peer-reviewed literature can be observed (Rowe 2019) which raises the question of the importance of posters for other means of scholarly communication. Thus far, studies conducted on the prevalence and dissemination of academic posters mostly focused on the impact and leverage of posters at conferences (Salzl et al., 2008; Rowe and Ilic, 2009; Wallengren Lynch, 2017). However, not a single study vetted the ramification of scientific posters on academic publishing, especially through scientometric data mining.

## Methods

For this study, a collection of posters had to be identified. This collection had to match several criteria.

1. The posters must be considered “published.” In order to be cited, we assume that a publicly accessible digital reproduction must be available.
2. Each poster must be assigned a Digital Object Identifier (DOI) to allow harvesting of citation counts.
3. The retrieved DOIs need to be registered in either Crossref or Datacite, since data from these DOI registration agencies are openly available and free to use.
4. A comprehensive analysis of posters requires a well curated and extensive collection.

Poster collections from the multidisciplinary open source repositories Zenodo and Figshare were found, each containing at least 5000 records (Zenodo: 7026, Figshare: 6018) as well as a low amount of mislabeled documents, which were validated manually in small data samples.

Figshare is a commercial internet based data storage focusing on academic research data management and research data dissemination. Figshare is maintained by Digital Science and was initially launched in 2011. Zenodo is an open digital archive developed by CERN and OpenAIRE.

The study takes into account the years 2016 until 2020 according to the date of publication on the platforms. We have decided to examine the years 2016–2020 because we can only query the complete range of posters in both repositories for these years due to API limitations. Also, we were not able to retrieve the actual publication date *via* API. Both APIs from Figshare and Zenodo only contain the date of publication on the platform. Accordingly, the date of publication on the platform may differ from the actual first publication. An important advantage of each repository is that research data can be filtered and sorted by various document types. For instance, it is possible to retrieve only posters from each repository, which is necessary for this inquiry.

In total, 13,044 records were retrieved and analyzed. Publication data was harvested *via* the OAI-PMH API of Figshare as well as by using Zenodo’s REST-API. Data was harvested between 11 October and 12 October 2021. OAI-PMH is a widely used protocol for harvesting metadata descriptions. A REST-API is a programming interface. It describes an approach for communication between client and server in networks.

As stated above, each record should be assigned a unique identifier (DOI). We matched those DOIs with Crossref, Dimensions and DataCite to obtain citation counts for each poster. DataCite is a non-profit organization that organizes the allocation of DOIs for academic institutions. Another similar agency is Crossref. Crossref also operates as a non-profit, run by Publishers International Linking Association Inc. (PILA). Both also provide bibliographic and citation data. Dimensions is another comprehensive citation database. Both poster collections predominantly include posters with DOIs assigned by DataCite. Only a small fraction of DOIs originate from other registration agencies than DataCite.

We used a simple Python script to retrieve and harvest posters from Zenodo and Figshare and also for matching DOIs with the mentioned citation databases. We considered non-matching DOIs as zero-cited publications/posters. For posters that are available in both Figshare and Zenodo, we extracted the DOI prefix to indicate the affiliation to a repository. In addition, we eliminated duplicate DOIs with a lower citation count than their counterparts. For instance, if citation counts for a single DOI were found in Dimensions (twice) and Crossref (once), we valued the Dimensions entry higher, since it found and indexed more citations. Therefore in this specific example, Crossref’s entry would be removed from the dataset. There were no cases of equal reference counts between citation sources in our dataset (except for DOIs that were found in both repositories).

According to Hyndman (2020) Figshare should have the same information as Dimensions, which it uses to provide citation data. For this study, we tested both APIs and we noticed data discrepancies between the provided citation counts looked up for a given DOI. Since the citation counts we gathered *via* the Figshare API were much more comprehensible, we decided to forego the use of the Dimensions API in this study.

## Result

Overall, citations could only be found for 1% of the posters in our dataset, a total of 137 posters. The number of posters as well as the number of cited posters for each data repository we examined is shown in Table 1.

**TABLE 1:** Number of posters for each data repository.

Source	Number of posters	Number of cited posters
Figshare	6,018	58 (1.0%)
Zenodo	7,026	79 (1.1%)

**FIGURE 1:**
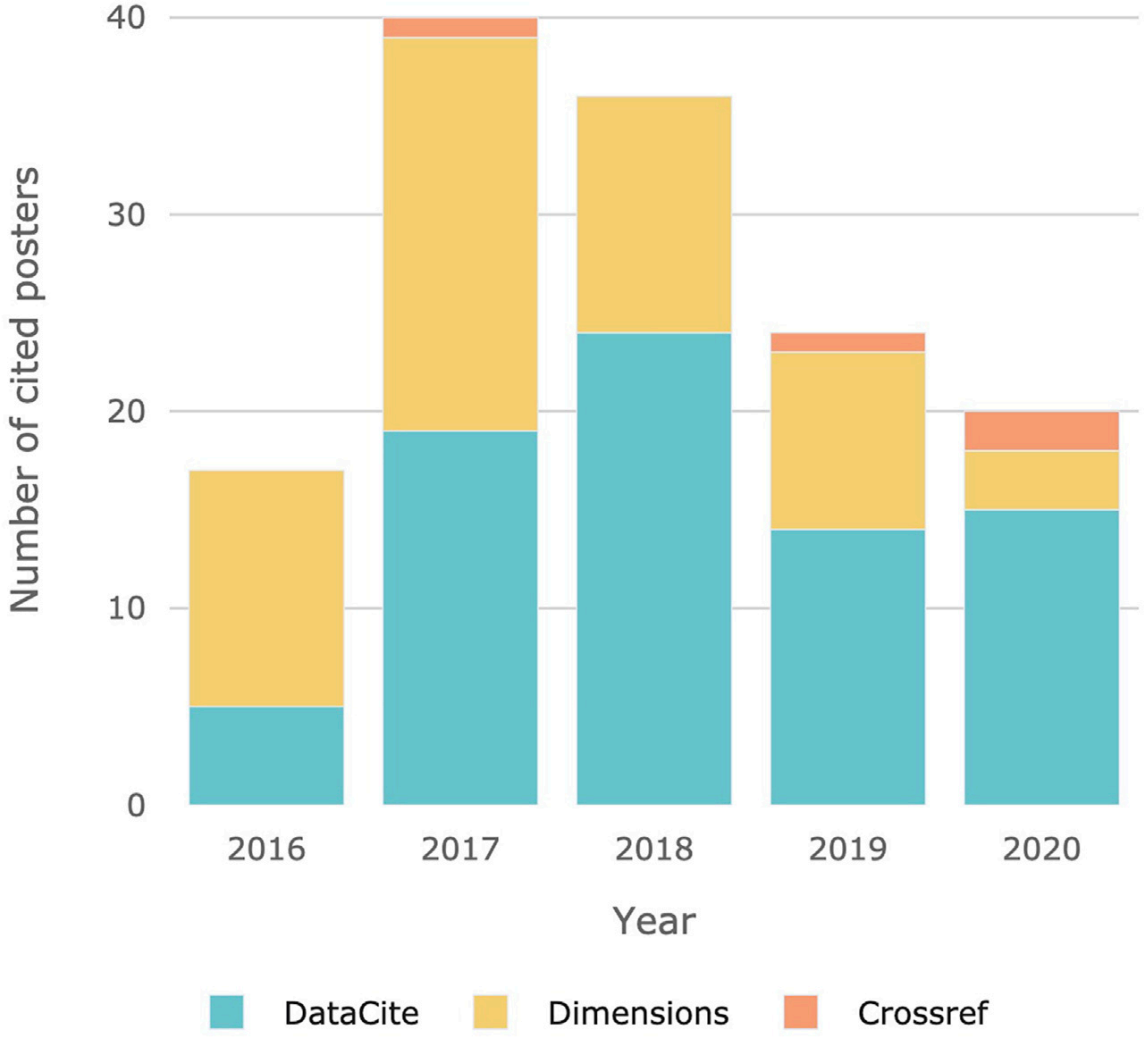
Cited posters for each data repository.

Figure 1 shows the number of posters cited over the years 2016–2020 (for Zenodo and Figshare). While a considerable increase in citations can be measured between 2016 and 2017 the citation rate decreases continuously from 2017 to 2020. In 2016, the lowest number of citations were measured, whereas in 2017 most of the posters were cited. Most citations were found in DataCite (77). Only four citations are from Crossref and 56 from Dimensions. As noted above, we only considered the citation source with the highest citation count for each DOI. With that said, 55% of citations are recorded in DataCite and 40% from Dimensions. Additionally, we found a certain amount of false positives, that is, documents categorized as posters that were actually other publication types and therefore mislabeled. In total, 7% of DOIs in the dataset are incorrectly categorized as posters. We excluded these DOIs for this analysis to receive more comprehensive outcomes.

**FIGURE 2:**
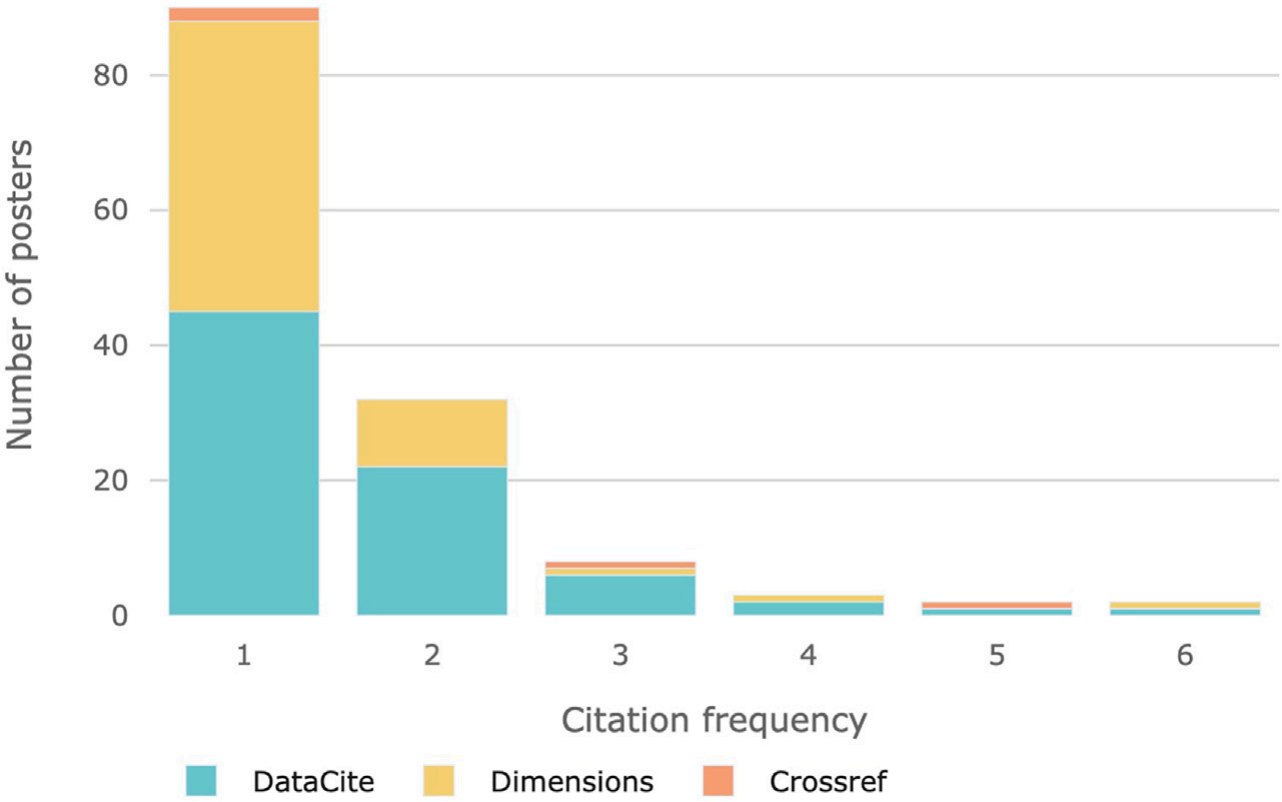
Cited posters to citation frequency.

As Figure 2 shows, most academic posters are only cited once according to data taken from our selected citation databases. However, a small fraction of posters were cited multiple times up to five, in two cases up to six times.

## Discussion

For our analysis we didn’t differentiate between self-citations and other citations, as we wanted to investigate whether posters are cited at all. We did not map posters to disciplines or fields of research, either. The question, how posters are cited, and if poster citation behaviour is different from one discipline to the other, is fertile ground for further research.

The method and the data we used have some clear limitations. First of all, the data: To our knowledge, there is no data source that provides a large poster collection with sufficient data in good metadata quality. The data sources we selected offer the possibility of uploading poster collections or individual posters, and there is little to no curation effort on the platforms we chose. We therefore have a non-representative sample, which nevertheless provides a good first indication for answering our question of whether posters are cited.

Our study shows that academic posters, despite their significant role in scholarly communication, are sparsely cited. This may indicate a lack of accessibility and findability as well as a low reputation of posters as a citation source among researchers and scientists. Apparently, it is generally more accepted to cite a paper related to a poster since it is a widely recognized format.

Besides, not every data repository stores posters or labels them correctly. In this study, for instance, we recognized that approximately 7% of posters in Figshare and Zenodo were actually mislabeled. Also, it seems that oftentimes related posters and papers are named similarly so that it is harder to find the original related poster. Furthermore, our data reveal no specific gap between the number of citations for posters of different scientific disciplines, although this was not investigated in depth.

For more conclusive results, we contemplate matching DOIs with Scopus, Microsoft Academic and Google Scholar to retrieve more balanced citation data due to higher recall. Nevertheless, when trying to receive data from these services one will inevitably be faced with some obstacles e.g. restricted access or no public API.

Another way to study the leverage of academic posters would be to take altmetrics into consideration. With respect to our research question, posters could for example also be cited in micro-publications like blogs, tweets and academic social platforms such as Academia.edu and Researchgate. Eventually, this study could be an impulse to encourage researchers and analysts to start further studies in this particular research domain, since posters are playing a crucial role at academic conferences.

## Data Availability

The datasets generated and analyzed for this study can be found on GitHub: https://github.com/naustica/poster_citations.
